# The effect of pumpkin seed cake and ground cloves (*Syzygium aromaticum*) supplementation on gastrointestinal nematode egg shedding in sheep

**DOI:** 10.1051/parasite/2021076

**Published:** 2021-12-06

**Authors:** Jožica Ježek, Karmen Mirtič, Nina Rešetič, Jaka Jakob Hodnik, Aleksandra Vergles Rataj

**Affiliations:** Veterinary Faculty, University of Ljubljana Gerbičeva 60 1000 Ljubljana Slovenia

**Keywords:** Sheep, Pumpkin seed cake, Clove, Faecal egg count, Helminths, Feed supplement

## Abstract

The aim of this study was to evaluate the effect of feed additives (pumpkin seed cake and cloves) on the egg excretion of gastrointestinal nematodes (GIN) in sheep. Thirty ewes naturally infected with GIN were randomly selected from a flock and assigned to the following groups of 10 animals each: clove group (received 1.8 g ground cloves/ewe/day, for 7 days), pumpkin seed cake group (200 g pumpkin seed cake/ewe/day, for 7 days) and control group. Before starting the study, on day 0, and 5 days after the 7-day supplementation, on day 12, the body condition and FAMACHA scores were assessed and individual faecal egg counts (FEC) were performed. The mean body condition and the FAMACHA scores did not change significantly between day 0 and 12 with the exception of a significantly deteriorated FAMACHA score in the clove group. The percentage reduction of FEC was 40.7% on day 12 in the clove group and 52.9% in the pumpkin seed cake group. In the control group, FEC increased by 8.7%. A coproculture of faecal samples from four of the most infected animals on day 0 revealed *Trichostrongylus* spp. larvae L3 in all four selected ewes, *Ostertagia* spp. and *Cooperia* spp. in three and *Haemonchus contortus* in one ewe. These results are promising and encourage further studies aimed to evaluate the possibility that these plant supplements could be a complementary method for parasite control, thus reducing the need for chemotherapy.

## Abstract (fr)

Le but de cette étude était d’évaluer l’effet des additifs alimentaires (tourteaux de graines de citrouille et clous de girofle) sur la production d’œufs par les nématodes gastro-intestinaux (NGI) chez le mouton. Trente brebis naturellement infectées par des NGI ont été sélectionnées au hasard dans un troupeau et réparties dans les groupes suivants de 10 animaux chacun : groupe clou de girofle (1,8 g de clous de girofle moulus / brebis / jour, pendant 7 jours), groupe tourteau de graines de citrouille (200 g de tourteau de graines de citrouille / brebis / jour, pendant 7 jours) et groupe témoin. Avant de commencer l’étude, au jour 0, et cinq jours après la supplémentation de 7 jours, au jour 12, l’état corporel et les scores FAMACHA ont été évalués et des dénombrements d’œufs fécaux (DOF) individuels ont été effectués. L’état corporel moyen et les scores FAMACHA n’ont pas changé de manière significative entre le jour 0 et le jour 12 à l’exception d’un score FAMACHA significativement détérioré dans le groupe clou de girofle. Le pourcentage de réduction du DOF était de 40,7 % au jour 12 dans le groupe clou de girofle et de 52,9 % dans le groupe tourteau de graines de citrouille. Dans le groupe témoin, le DOF a augmenté de 8,7 %. Une coproculture d’échantillons fécaux de quatre des animaux les plus infectés au jour 0 a révélé des larves L3 de *Trichostrongylus* spp. chez les quatre brebis sélectionnées, *Ostertagia* spp. et *Cooperia* spp. dans trois et *Haemonchus contortus* dans une. Ces résultats sont prometteurs et encouragent de nouvelles études visant à évaluer la possibilité que ces suppléments végétaux puissent être une méthode complémentaire de contrôle des parasites, réduisant ainsi le besoin en chimiothérapie.

## Introduction

Grazing animals, particularly small ruminants, are frequently infected by gastrointestinal nematodes (GIN), resulting in reduced productivity and increased mortality [38]. For many years, parasitic diseases in ruminants have been controlled by the use of broad-spectrum and commonly available anthelmintics. However, the continuous and inappropriate use of anthelmintic drugs has led to a loss of effectiveness. Anthelmintic resistance has been reported in many European countries [16, 36] and worldwide [38].

The increase in resistance of GIN of small ruminants to conventional anthelmintic drugs implies the need for alternative strategies to reduce the parasite burden in the animals and the number of infective larvae in the pasture [18]. Alternative strategies are necessary not only because of resistance to anthelmintic drugs, but also due to consumer demand for reduced use of drugs in meat animals and for lower chemical residues in food and the environment [7]. One of the options could be to feed animals with plants that are not only a source of nutrients but also contain bioactive substances (tannins, etc.) with potential anthelmintic effects [8, 17–21, 29]. When discussing whether a drug or a medicinal plant extract is effective against parasites *in vitro*, it must be borne in mind that in order to be medically useful, such a substance must be bioavailable and should not intoxicate the patient [47].

Pumpkins belong to the Cucurbitaceae family, which comprises 130 genera and about 1000 species [9]. Among them are several medically important genera, one of which is *Cucurbita* [13]. *Cucurbita pepo* comprises three subspecies, *C. pepo* L. subsp. *fraterna*, *C. pepo* L. subsp. *ovifera* and *C. pepo* L. subsp. *pepo* [9, 34]. *Cucurbita pepo* L. subsp. *pepo* var. *oleifera* is an oil pumpkin with thin-coated seeds [43] and is cultivated for its seeds and seed oil. Pumpkin seed cake used in our study is a by-product in oil production. Cucurbit plant, which is native to the American continent, has been used in folk medicine worldwide for the treatment of gastrointestinal diseases and intestinal parasites, as well as other clinical conditions [37]. Pumpkin seeds (*Cucurbita pepo* L.) contain saturated and unsaturated fatty acids such as palmitic, palmitoleic, stearic, oleic, linoleic and α-linolenic acid and are rich sources of protein. They contain secondary metabolites such as terpenoids, quinones, saponins, steroids, phenols, tannins, alkaloids (berberine), cucurbitine, and palmatine [14, 17]. These secondary metabolites might have anthelmintic action [17]. Ethanol pumpkin seed extract, and hot and cold water pumpkin seed extracts significantly influenced the survival of L1 and L2 *Heligmosomoides bakeri* larvae compared to a negative control *in vitro*. An *in vivo* study showed that ethanol *C. pepo* seed extract administered to mice effectively reduced both the faecal egg count and the number of adult stages of *H. bakeri* [17]. Pumpkin seed treatment resulted in a significant reduction in the initial number of faecal worm eggs in sheep and showed a potential for parasite control [41].

The clove (*Syzygium aromaticum* L.), a member of the Myrtaceae family, is an aromatic tree native to the Moluccas and southern Philippines, but currently grown in many tropical areas including Africa, South America, Indonesia, Malaysia and Sri Lanka [40, 48]. It is an evergreen tree, 10–20 m tall, with spear-shaped leaves and cluster-like, yellowish flowers. Dried flower buds are commonly used in cooking, pharmacy, perfumery and cosmetics [40]. The main ingredient (up to 20%) is essential oil, characterised by the presence of eugenol, eugenol acetate and α- and β-caryophyllene [48]. Eugenol is the main bioactive compound [12]. From an aqueous acetone extract of dried flower buds of *Syzygium aromaticum* 18 hydrolysable tannins were isolated [4]. Anthelmintic effects of hydrolysable tannins have been evaluated in several studies [1, 11]. The clove is an important medicinal plant due to its broad spectrum of pharmacological effects (antioxidant, antimicrobial, antinociceptive, and antiviral), and has been used in traditional applications for centuries. Its medicinal uses are described in the literature [12]. Ethanol extract (0.5 mg/mL) of *Syzygium aromaticum* inhibited energy metabolism in the nematode *H. contortus in vitro*, resulting in reduced production of ATP, which leads to death of the parasite [29]. Clove extract showed *in vitro* dose- and time-dependent ovicidal and anthelmintic effects on *H. contortus* [8] and *Fasciola gigantica* [28].

The aim of the present study was to investigate the efficacy of diets with pumpkin seed cake and cloves on the egg excretion of GIN in sheep naturally infected with GIN under usual farming conditions.

## Materials and methods

### Ethics

The study was conducted at a sheep farm in central Slovenia between December 2019 and January 2020. All procedures complied with relevant Slovenian legislation (Animal Protection Act, Official Gazette of the Republic of Slovenia, No 38/2013).

### Study animals

There were 120 sheep of the breed Jezersko-Solčava (autochthonous Slovenian breed) grazing on the farm from spring to autumn. This breed is annual poliestric and is used for meat production; the body weight of ewes is between 65 and 80 kg. After the grazing season, the animals were kept in a barn during the winter months. During the entire study, the animals were kept indoors. The winter ration consisted of hay (two thirds) and grass silage (one third), which was provided *ad libitum*. The sheep had unrestricted access to clean water.

The animals were not dewormed for 7 months prior to the study.

The study was performed on a total of 30 ewes aged between 1.5 and 13 years (median 3.8 years). Seven ewes lambed within 2 months before the beginning of the study, and others were pregnant. The ewes were randomly selected from the flock and randomly assigned to the following supplementation groups of 10 animals each: clove supplemented group, pumpkin seed cake supplemented group, and control group.

Groups supplemented with clove and pumpkin seed cake were kept in separate pens; the control group was kept with the flock.

### Diet

The group supplemented with cloves received 1.8 g of freshly ground cloves/ewe/day for 7 days in addition to the usual ration. The cloves were bought in a local shop.

The group supplemented with pumpkin seed cake (*Cucurbita pepo* var. *oleifera*) received 200 g pumpkin seed cake/ewe/day divided into two meals (morning, evening) for 7 days with the usual ration. The composition of the pumpkin seed cake is shown in Table 1.

**Table 1:** The composition of pumpkin seed cake.

Component	Content (%)
Moisture	6.04
Raw protein	57.0
Raw oils and fats	13.5
Raw fibre	3.50
Raw ash	9.43

Although the sheep were reluctant to eat cloves at the first day, they later got used to the taste and willingly ate the offered feed supplements.

The control group was fed with the usual ration. The owner received detailed instructions on how the animals in each group should be fed.

### Measurements

At the beginning of the study, on day 0, and 5 days after 7-day supplementation on day 12, body condition score (5-point scale) [26] and colour of conjunctivas (FAMACHA score) [5, 24] were assessed in each ewe. Body condition score was assessed with palpation of the loin region (muscling, fat deposition on spinous and transverse processes): score one means emaciated and score five means obese.

Faecal egg counts were performed on each study animal on days 0 and 12. Individual faecal egg counts were determined by using McMaster technique [49].

A coprological culture of faecal samples from the four most infected animals was performed on day 0 to get information about helminth genera present in the flock. Sheep faecal samples collected in plastic bags were incubated for 12 days, at a temperature of 26–30° C. L3 larvae were obtained by baermannization and identified using identification keys [44].

### Data analysis

Using the SPSS software package for Windows (version 22) [23], descriptive statistics for body condition score, FAMACHA score, and faecal egg count was calculated. To compare values of investigated variables between day 0 and day 12 for each group, a Wilcoxon signed-rank test for dependent samples was used. Faecal egg counts among the groups before and after supplementation were compared using an independent samples Kruskal-Wallis test and Dunett’s T3 post hoc test.

To determine the percentage of Strongylid eggs shedding reduction in samples after the supplementation, we calculated the mean number of eggs in the samples from each group before supplementation (T1) and after supplementation (T2), and used the equation [25, 27]:

$$\mathrm{Egg}\;\mathrm{reduction}\;\left(\%\right)=100\times \left(1-\left\lceil \frac{T2}{T1}\right\rceil \right).$$

The 95% confidence intervals were calculated with the eggCounts on-line analysis program [25, 46], using the Two samples paired model procedure (http://shiny.math.uzh.ch/user/furrer/shinyas/shiny-eggCounts/).

Eggs of other parasites and coccidian oocysts were found only in small numbers in some animals and were therefore not included in the statistical analysis.

## Results

### Body condition- and FAMACHA score

The results of the body condition assessments in clove-, pumpkin seed cake- and control group sheep are presented in Table 2. The mean body condition score values did not change significantly in any of the groups.

**Table 2:** Body condition score before (day 0) and after supplementation (day 12).

Group	Day 0 (mean)	Range	Day 12 (mean)	Range	*p*-value
Clove	2.7	2.0–3.5	2.7	1.5–4.0	1.000
Pumpkin seed cake	3.0	2.0–4.5	2.9	1.5–4.0	0.317
Control	2.5	2.0–3.0	2.4	2.0–4.0	0.564

The results of conjunctiva colour score (FAMACHA) in the clove-, pumpkin seed cake- and control group sheep are presented in Table 3. The FAMACHA score values increased slightly on day 12 in all three groups, with a significant difference found only in the clove group.

**Table 3:** FAMACHA score before (day 0) and after supplementation (day 12).

Group	Day 0 (mean)	Range	Day 12 (mean)	Range	*p*-value
Clove	2.3	1.0–4.0	2.8	2.0–4.0	0.025
Pumpkin seed cake	2.2	1.0–3.0	2.6	1.0–4.0	0.217
Control	2.5	1.0–4.0	2.8	2.0–4.0	0.366

### Faecal egg counts

**Figure 1:**
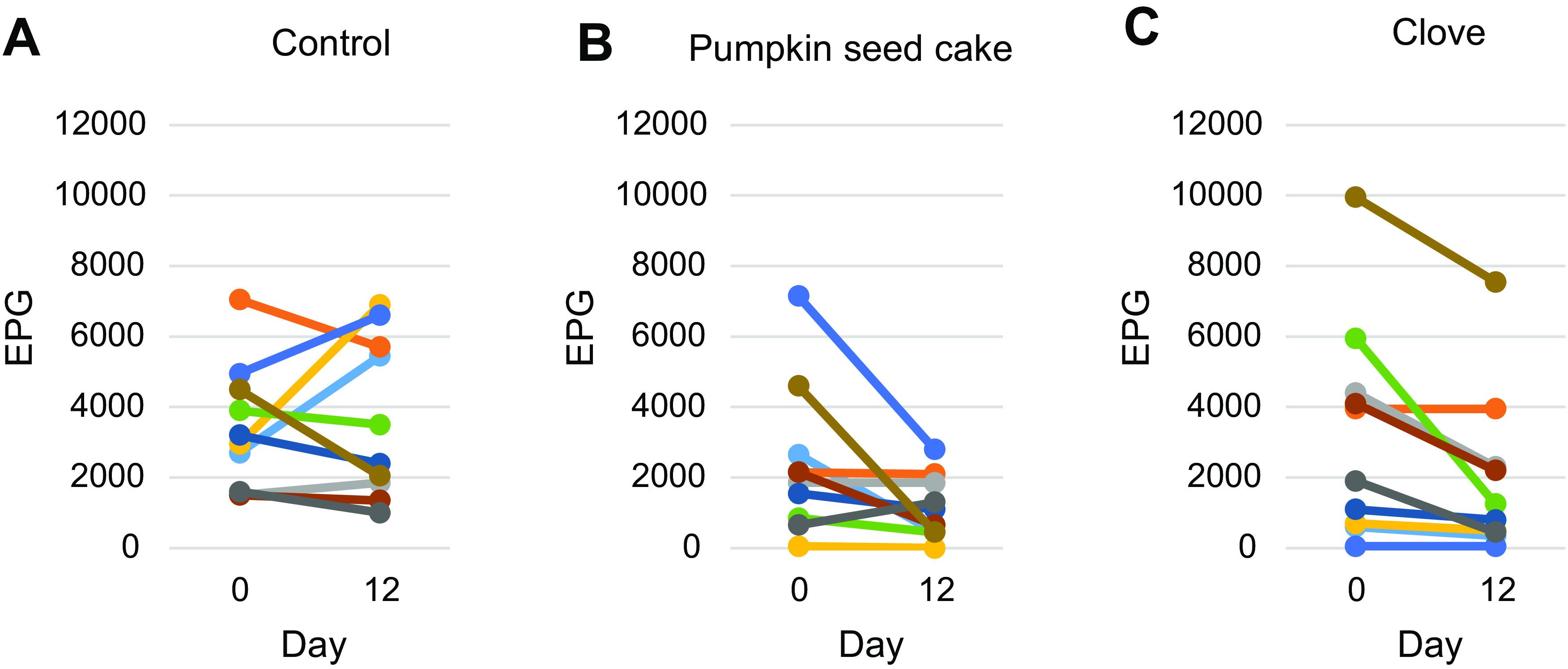
The results of Strongylid faecal egg counts in the control (A), pumpkin seed cake (B) and clove (C) group (the lines represent individual ewes).

The results of faecal egg counts in the clove-, pumpkin seed cake- and control group sheep are presented in Table 4 and Figure 1. In the clove group, FEC decreased in eight ewes after the supplementation, while it remained unchanged in two ewes. The percentage decrease of FEC was 40.7% on day 12, with a 95% confidence interval of 0.32–0.47. In the pumpkin seed cake group, FEC decreased in eight ewes after the supplementation, increased in one, and remained unchanged in one ewe. The percentage reduction of FEC was 52.9%, with a 95% confidence interval of 0.45–0.60. In the control group, FEC decreased in six ewes after the supplementation, and increased in four ewes. The percentage increase of FEC was 8.7%.

**Table 4:** Strongylid faecal egg counts in eggs per gram (EPG) and percentage reductions in faecal egg counts (FECR).

Group	Day 0 – EPG (mean)	EPG (range)	Day 12 – EPG (mean)	EPG (range)	FECR (%)	*p*-value
Clove	3270	50–9950	1940	50–7550	40.7	0.012
Pumpkin seed cakes	2365	50–7150	1115	0–2800	52.9	0.038
Control	3385	1500–7050	3680	1000–6900	−8.7	0.959

The comparison of FEC between the groups before supplementation showed insignificant (*p* = 0.480) differences, and after supplementation, the differences between the groups were statistically significant (*p* = 0.020), the pumpkin seed cake group differed significantly from the control group (*p* = 0.018).

### Coprological culture

*Trichostrongylus* spp. larvae L3 were found in all four selected ewes. L3 larvae of the genera *Ostertagia* spp. and *Cooperia* spp. were found in three ewes and only a few *Haemonchus contortus* larvae were found in one ewe.

## Discussion

In this study, we explored the *in vivo* effect of pumpkin seed cake and clove supplements on nematode egg excretion in naturally infected ewes in farm conditions. In several published studies, there was evidence of anthelmintic properties of pumpkin seed and cloves *in vitro* and *in vivo* [8, 17, 28–31, 41]. Diet can influence the host’s ability to cope with the consequences of parasitism in different ways. It can increase the host’s ability to cope with the negative consequences of parasitism (resilience), it can improve host resistance, and it can directly affect the parasite population via ingestion of anthelmintic compounds [10].

To evaluate performance and detect clinical anaemia in the ewes with on-farm methods, we assessed body condition [26] and FAMACHA score [24]. The body condition of the ewes remained almost the same during supplementation in all groups studied. In most ewes, the body condition was within the recommended range of 2.0–3.5 [26]. Despite the high nutritional value of pumpkin seed cake (rich in protein), body condition score values did not improve in the group of ewes receiving this supplement. The ewes were fed with pumpkin seed cake for only seven days. The period between the two evaluations may have been too short for a noticeable improvement in body condition and to detect the differences in the mean body condition score between the groups. To change the body condition score in a sheep by one unit, the animal needs to gain 3.3–16.0 kg live weight depending on the breed and category of the animal (lamb after weaning, lactating ewe, dry ewe) [26]. Perhaps the weighing of the animals in our study could have led to different results, as we would have detected even small changes in live weight.

The mean conjunctiva score (FAMACHA) deteriorated slightly in all groups during the study. Although the results of the parasitological examination after supplementation were favourable in both supplemented groups, this may be partly due to the possible presence of hematophagous nematode species and the fact that the newly formed erythrocytes take some time to mature and become fully functional. Thus, they only appear in the bloodstream after 5 days [35]. Despite the high initial number of faecal nematode eggs in many ewes, the conjunctival score values in most ewes did not indicate serious anaemia; mean score values in all groups were between 2.2 and 2.5. Anaemia is considered at eye score of ≥3 [24]. This is consistent with the results of coproculture, where most of the larvae found were not from hematophagous nematode species.

The faecal egg counts changed differently between the groups after supplementation. In the control group, the average number of eggs increased (but the difference between day 0 and 12 was not statistically significant), which was to be expected since the animals did not receive any preparations or additives that would be effective against parasites. The juvenile forms of the parasites were able to mature and even started to produce eggs on their own. The number of eggs did not increase in some ewes, suggesting that some other factors (such as physiological status, etc.) might influence egg excretion by the parasites.

In the group of ewes that received a pumpkin seed cake supplement, the faecal egg count after supplementation decreased by an average of 52.9%. The decrease in the faecal egg count could be attributed to the active ingredients in pumpkin seed cake and partly to the high protein content (57% crude protein). It has been shown that adequate protein supplementation can help animals with pre-existing severe gastrointestinal worm infection by preventing or reducing clinical signs of *H. contortus* infection making pathological changes less pronounced [42]. A protein-rich diet in dairy goats resulted in a significantly lower faecal egg count, suggesting that resistance was enhanced by protein supplementation [15].

Anthelmintic properties in pumpkins are primarily attributed to the secondary metabolite cucurbitin in pumpkin seeds. This accelerates the excretion of the parasites from the host by weakening their ability to attach to the gastrointestinal wall [2], and also causes degenerative changes in the reproductive organs of the parasites [6]. Triterpenoid cucurbitacin also has anthelmintic activity [37]. Pumpkin seeds also contain tannins which have shown anthelmintic properties in several studies [19–21, 32, 33, 39] and could have contributed to decreased faecal egg counts. In another study, 4-month-old Merino lambs were infected with *H. contortus* larvae and treated with pumpkin seeds for two weeks. The treatment resulted in a 65.5% decrease in the initial faecal egg count, which, however, increased back to baseline as soon as the animals came off the treatment. The authors concluded that pumpkin seeds have a potential to control parasites by influencing parasite fertility [41]. In an experiment in which naturally infected goat kids were supplemented with ground pumpkin seeds (5 g/kg body weight), the researchers observed no effect on faecal egg count, but the treatment did affect the packed cell volume (*p* < 0.01), which was greater in the treated group than in the control group [31]. Although the faecal egg counts were similar among treatments, no animals treated with pumpkin showed clinical signs of infection (diarrhoea, bottle-jaw, anaemia) and were therefore not dewormed [31]. Pumpkin seeds have also shown anthelmintic effects in other animal species, e.g. in poultry [2] and in mice [3], where there was a reduction in the number of adult parasites and also in the number of excreted eggs. Bauri et al. [6] described the effect of pumpkin seed extract on *H. contortus* under *in vitro* conditions where the motility of adult parasites was reduced. Grzybek et al. [17] monitored blood and urine parameters as well as histopathology in pigs and rats during long-term feeding with pumpkin seed extract and found no abnormalities. Pumpkin seed cakes are a tasty feed for animals; they are easily accessible to farmers and can be used as high-protein concentrates in the diet. Not only are they a promising alternative to the use of antiparasitic drugs, but they can also be a source of protein. Results from Strickland et al. [41] also suggest that lambs can cope with high parasite loads in good nutrition and are still productive in the early stages of infection.

In the group of sheep supplemented with ground cloves, the number of strongylid eggs per gram of faeces decreased by 40.6% on average. The decrease in the number of excreted eggs can be attributed to bioactive substances in cloves (alkaloids, glycosides, essential oils, tannins, etc.), in particular eugenol [48], which is thought to act on the cuticle of the parasite, and tannins, which are known to have anthelmintic properties [19–21, 32, 33, 39]. Charitha et al. [8] investigated the *in vitro* effect of an acetone extract from cloves on egg hatching and on adult motility in *H. contortus*. The egg hatch test and adult motility assay revealed significant anthelmintic properties against *H. contortus*, namely that at a concentration of 10.0 mg/mL within 2 min after exposure, 100% mortality of the worms was achieved. The worm-killing effect of clove extracts could be attributed to their strong corrosive effect on the cuticle and tegument of helminths. In addition, clove extract inhibited the energy metabolism of *H. contortus* by inhibiting fumarate reductase and succinate dehydrogenase activity, which prevented the parasite from residing in the abomasum and being expelled by the host [29]. Manoj Dhanraj and Veerakumari [30] observed the effect of clove ethanol extract on acetylcholinesterase activity and motility in the paramphistome *Cotylophoron cotylophorum*. The results of these studies indicate a similar mode of action of the clove on helminths as with some anthelmintic drugs (energy metabolism, muscle paralysis).

Clove essential oil is generally recognised as a safe substance when consumed in quantities lower than 1500 mg/kg [12]. The administration of standardised polyphenolic extract of clove buds (Clovinol) to Wistar rats did not result in toxicologically significant changes in clinical observations or behaviour compared to the untreated control group of animals, ophthalmological examinations, body weight, organ weight, feed and water intake, urinalysis, haematological and clinical biochemical parameters, which all point to the no observed-adverse-effect level at the highest dose tested (1 g/kg body weight per day) [45].

No side effects were observed in our study. Cloves are easily accessible and easy to dose. The farmer told us that the sheep did not want to eat them on the first day, probably because of the specific taste and smell, but later they had no problems consuming cloves.

The results of this study suggest that pumpkin seed cake and cloves may have the potential to reduce faecal egg counts of GIN.

*In vivo* studies on the effect of pumpkin seeds and cloves on the excretion of gastrointestinal nematode eggs are sparse in small ruminants. To the best of our knowledge, no previous study has investigated the effect on gastrointestinal nematode eggs excretion of pumpkin seed cake or cloves added to the diet of naturally infected sheep. The results obtained are promising for the potential use of pumpkin seed cakes and cloves added to the diet of sheep for GIN control. Moreover, pumpkin seed cakes and cloves are available to farmers and are easy to use as a feed supplement and could therefore become a part of an integrated approach to parasite control, through which treatment with anthelmintic drugs could be reduced. Nonetheless, this study shows some weaknesses. Firstly, identification and description of the plant secondary metabolites were not performed, considering that their concentration may be highly variable [22, 37]. Additionally, the different diets were not analysed for macronutrients and were not balanced among groups, which could confound the real anthelmintic effects of the plant supplements. The feeding trial with pumpkin seed cake and cloves lasted for a very short period, while a longer period might have resulted in a greater anthelmintic effect. Therefore, further studies are needed to confirm the results obtained in this study and to evaluate the real possibility that these plant supplements could be used as a new method for the control of small ruminant GIN in the future. The exact mechanisms of their mode of action should be addressed in further research and the effect on different parasite species should be investigated as well. Further research would be needed on combined use of pumpkin seed cake and cloves to investigate their potential synergistic effects on GIN in small ruminants. The study should be repeated on different breeds of sheep in uniform groups, at different seasons and various basic diets (grazing, winter diet) in order to generalise the findings to any animal species.
